# An alien intermediate snail host in Malawi - *Orientogalba viridis* (Quoy and Gaimard, 1832) - A new concern for schistosomiasis transmission in Africa?

**DOI:** 10.1016/j.ijppaw.2024.100919

**Published:** 2024-03-01

**Authors:** A. Juhász, C. Nkolokosa, E. Kambewa, S. Jones, L.J. Cunningham, P. Chammudzi, D. Kapira, G. Namacha, D. Lally, S.A. Kayuni, P. Makaula, J. Musaya, J.R. Stothard

**Affiliations:** a Department of Tropical Disease Biology, Liverpool School of Tropical Medicine, Liverpool, L3 5QA, UK; b Institute of Medical Microbiology, Semmelweis University, H-1089, Budapest, Hungary; c Malawi-Liverpool-Wellcome Programme, Blantyre, Malawi

**Keywords:** Fascioliasis, Gastropoda, Invasive species, Lymnaeidae, Trematodiasis

## Abstract

The freshwater amphibious snail *Orientogalba viridis* commonly occurs in eastern Asia, on certain Pacific islands and more importantly has recently dispersed into Europe. Since this snail is now considered an invasive species, its distribution is of growing parasitological interest as an alien intermediate host for various trematodes, particularly liver flukes. As part of ongoing surveillance for snail-borne diseases in Malawi, a population of *O. viridis* was first observed in May 2023, alongside an alarming presence of a human schistosome cercaria. This snail population later underwent detailed morphological characterisation with both snail and parasite identities confirmed upon DNA barcoding. This seminal observation triggered more extensive local snail surveys, finding 3 further populations in separated rice paddies, with further field-caught snails (n = 465) screened for infection and a selection used for repeated experimental challenges with miracidia from *Schistosoma haematobium* and *Schistosoma mattheei*. Although no field-caught (and experimentally exposed) snail was seen to shed schistosome cercariae, molecular xenomonitoring for schistosomiasis provided tangible evidence of putative transmission potential. Our first report of *O. viridis* here in Malawi, and more broadly in Africa, flags a need for increased vigilance for this invasive species alongside local clarification(s) of its transmission potential for trematodiases of either medical and/or veterinary importance.

## Highlights

• This is the first report of the *Orientogalba viridis* freshwater snail species in Malawi and in Africa, denoting a transcontinental invasion from Asia.
• The taxonomic identification of the molluscs was confirmed by morphological and molecular analyses taking a total evidence approach.
• Experimental infection of *O. viridis* to *S*. *haematobium* and *S*. *mattheei*, with molecular xenomonitoring, evidenced some schistosome development.
• This alien snail may well be present in other parts of Africa and is perhaps already changing the transmission of trematodiases.

## Graphical abstract

**Figure:**
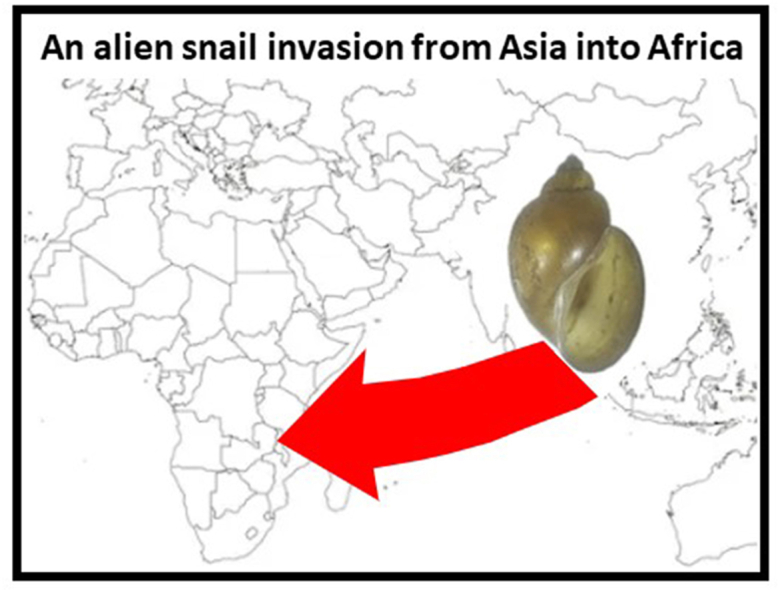

## 1 Introduction

The amphibious lymnaeid snail *Orientogalba viridis* (Quoy and Gaimard, 1832) has an ancestral Asian distribution (Aksenova et al., 2018) where today it plays a prominent role as local intermediate host for various trematodiases, foremost with human and animal fascioliasis (Correa et al., 2010; Liu et al., 2012; Mas-Coma S, et al., 2022). Following the report of Schniebs et al. (2017), *O. viridis* was first noted within rice paddies in Spain, thereafter this species was considered an invasive alien species to Europe (Schniebs et al., 2017). Its dispersion and invasion mechanisms from Asia and Australasia to Europe, however, remain unclear and speculative, though its strong connection with rice paddy cultivation might suggest strong links with agriculture.

Hitherto, *Orientogalba* Kruglov & Starobogatov, 1985 was unknown in Africa, however, identification and precise taxonomic status of this species is problematic. For example, to the untrained eye, snails may resemble other small juvenile lymneid species such as *Galba truncatula* (Müller, 1774) or *Radix natalensis* (Krauss, 1848). The latter has a pan-African distribution and is a major intermediate snail host for *Fasciola gigantica* (Cobbold, 1856); while the former snail species is geographically restricted to high altitudes (Brown, 1994; Mahulu et al., 2019). Of note following Aksenova et al. (2018), *O. viridis* was previously placed within the genus *Austropeplea* (Ponder and Waterhouse, 1997), though *Orientogalba* (with *Viridigalba* Kruglov and Starobogatov, 1985 a synonym) is now the preferred nomenclature. Indeed, accurate identification of Lymnaeid snails can be confusing and needs consideration of morphological and molecular evidence, the latter approach may take use of DNA barcoding as the database of sampled populations accrues (Schniebs et al., 2011; Vinarski et al., 2016).

As part of ongoing surveillance for snail-borne diseases in Malawi, a population of *O. viridis* was unexpectedly encountered in Chikwawa District, Malawi, alongside the alarming presence of a human schistosome cercaria. Our report fully describes this encounter and our later attempts to clarify the local distribution of this alien snail species and its potential role(s) in transmission of trematodiasis.

## 2 Materials and methods

### 2.1 The first encounter with *Orientogalba*

**Fig. 1:**
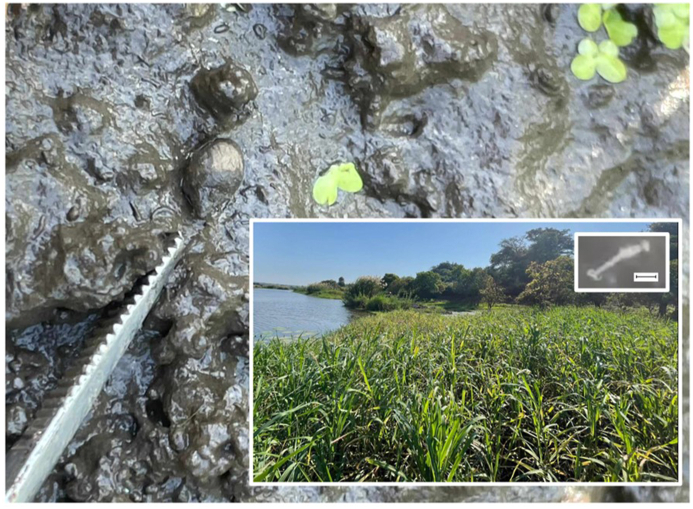
Site photo of the location where *O. viridis* was first encountered. Numerous snails were found on mud within the rice paddy [large inset] and, of particular note, a single schistosome cercaria of *S. haematobium* was observed upon microscopy and photographed [small inset] and confirmed by DNA barcoding. This rice paddy was immediately adjacent to a small oxbow lake, where children were seen swimming and numerous *Bulinus* were found but these snails were not observed to shed schistosome cercariae at the time of first survey in May 2023.

In May 2023, following a general malacological survey for intermediate snail hosts of schistosomiasis in Chikwawa District, Malawi, Central Africa, an unusual lymnaeid, superficially resembling *Galba* spp., was first noted at (S16.02528°, E34.82106°) within a spring-fed rice paddy. Numerous snails were observed on moist mud in between plants (Fig. 1). In total, 12 snails were collected, then placed in 100 ml of mineral water for inspection of shedding trematode cercariae which, later in that day, revealed an alarming presence of a single motile schistosome cercaria. This cercaria could not be readily explained as a contaminating artefact from environmental water, and likely originated from the snails themselves, though we acknowledge a possibility it may have been adhered to a shell. This larva was immediately harvested unto an FTA® card, subjected to DNA barcoding and was later confirmed as *Schistosoma haematobium* using protocols described by Webster et al. (2013). Inspecting surviving snails (n = 8) the following day for shedding cercariae did not yield any further evidence of patent infection(s).

**Fig. 2:**
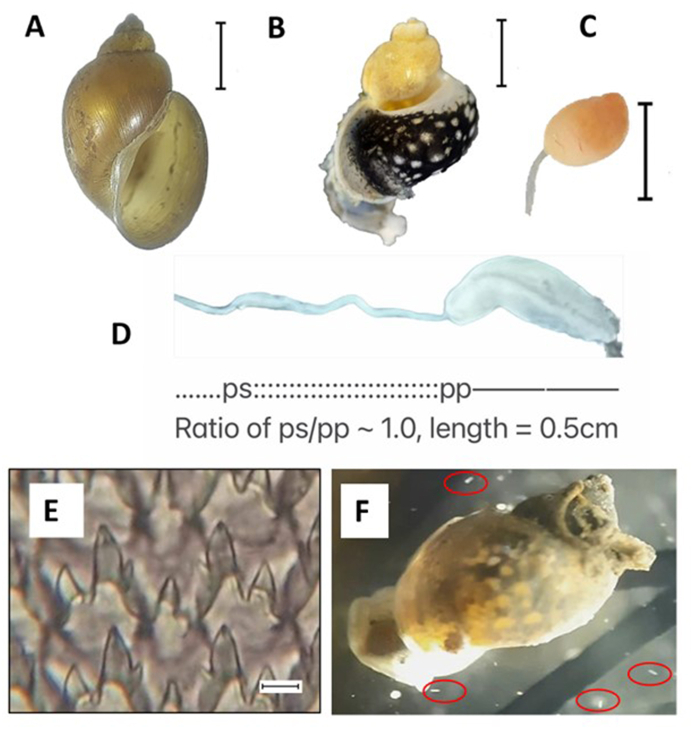
A) Shell, scale bar: 3 mm; B) mantle pigmentation, scale bar: 3 mm; C) bursa copulatrix, scale bar: 2 mm; D) penis, ratio of penis sheath (ps)/preputium (pp) ∼1, overall length 0.5 cm; E) radula, scale bar: 10 μm; F) experimental infection with *S. haematobium* with miracidia highlighted within red elipses with unaltered swimming around the snail's body. (For interpretation of the references to colour in this figure legend, the reader is referred to the Web version of this article.)

Alerted to this alarming concern for schistosomiasis transmission, in July 2023, 465 snails were collected around the edges of the same rice paddy. Water temperature was 26.0 °C, pH was 9.5, conductivity (uS) was 737.5 and total dissolved salts (ppm) was 370.5. Collected snails were placed in mineral water for inspection of shedding of trematode cercariae. In total, 25 dissected snails were euthanized by emersion in boiling water for 3 min, loosening the columellar muscle, allowing the soft body to be extracted from the shell with hooked metal forceps. The empty shells were cleaned, dried and preserved at room temperature for shell morphometrics. Snails macroscopically resembled *Orientogalba* spp.. Under the microscope, all dissected snails proved not to be infected with trematodes and were fully developed adults, according to the maturity of their genital organs. The genital structure was dissected out of the soft body and measured using a stereo microscope (Fig. 2A, B, C, D). The buccal mass was dissected out and digested in lactic acid for 48 h to free the radula from snail tissues and then examined under the compound microscope (Fig. 2E). A total evidence approach was taken to assess each character against the current lymnaeid literature, as there is no reliable species-specific morphological key.

### 2.2 Molecular identification of snails

To augment morphological evidence, a molecular taxonomic confirmation was undertaken. The mitochondrial cytochrome *c* oxidase subunit I (cox1) gene was amplified resulting in a single 600 bp sequence. Genomic DNA was extracted from the foot muscles of 71 specimens using the hexadecytrimethlammonium bromide method (Abbasi et al., 2007) followed by phenol/chloroform DNA extraction and ethanol precipitation. For amplification of the Folmer region of the *coxI* gene, PCR with primers: Folmer F: 5′-TTTTTGGWGTTTGATGTGG-3′ and Folmer R: 5′-TAAACTTCAGGGTGACCAAAAAATCA-3′(Folmer et al., 1994) was used. PCR products of ≈670 bp were detected from all 9 samples sent for Sanger sequencing, with nucleotide sequence consensus determined in both directions. A consensus sequence has been deposited in GenBank (accession number: OR793173.1).

### 2.3 Experimental snail infection

To clarify this snail's putative local role in schistosomiasis transmission, two experimental challenges were performed using miracidia of *S. haematobium* and *Schistosoma mattheei* Veglia and Le Roux, 1929. Respective miracidia were obtained from Mthawira village community (S35.30135°, E16.85423°), Nsanje District and from cattle from Montfort Mission (S34.82106°, E16.02528°), Mangochi District. Five groups of 48 *O. viridis* field-caught snails were each available for exposure; group-1 - a non-exposed control (to assess snail mortality), group-2, -3 and -4 were individually exposed to 1, 3 and 6 miracidia of *S. haematobium* whereas group-5 snails were individually exposed to 1 miracidium of *S. mattheei* following laboratory experimental protocols of Stothard and Rollinson (1997). Miracidial behaviour of each species was observed, upon close observation for 30 min under a stereo microscope (Fig. 2F).

After a 12 h period of exposure, groups were then separately maintained for 30 days in 5 transparent plastic boxes of 15cm × 15cm × 5 cm and fed *ad libitum*. The boxes were placed in a room under semi-natural conditions: a constant temperature of 25 °C. Every 2–3 days, snail mortality was monitored, with a selection of dead snails later checked by molecular xenomonitoring. On day 24 post-exposure (pe) and day 26 pe, day 28 pe and day 30 pe, all living snails were visually inspected for cercariae before a half of these were euthanized and checked for pre-patent infection by molecular xenomonitoring.

### 2.4 Molecular xenomonitoring - schistosomiasis and fascioliasis

For molecular xenomonitoring of schistosomiasis and fascioliasis, genus-specific and species-specific TaqMan® probes with real-time PCR were used. For schistosomiasis, first a genus specific primer-probe set was applied, followed by a species specific assay on certain snails. For the generic screen all 71 extracted genomic DNA samples were processed, this was performed in a 12 μL reaction volume using a Bio Molecular Systems MIC machine (Bio Molecular Systems, Queensland, Australia). Using the primers: SchGenF: 5′-GGTCTAGATGACTTGATYGAGATGCT-3′ and SchGenR: 5′-TCCCGAGCGYGTATAATGTCATTA-3′ with the probe Sch_gen: 5’-[Fam]TGGGTTGTGCTCGAGTCGTGGC[BHQ2]-3’. This was conducted at 5 min at 95 °C followed by 40 cycles of 5 s at 95 °C and 30 s at 60 °C. A species specific screen was performed on 13 samples to detect *S. haematobium* and *S. mansoni* as the snails were infected from miracidia originating from human urine and these two schistosomes are most common. Please note we regularly encounter ectopic *S. mansoni* eggs within human urine from this area. The assay took place as a duplex real-time PCR using the primers SchH_F: 5′-AATGAACATGAATGGCCGCA-2′ and SchH_R: 5′-ATGGGTTCCTCACCACTTAAACT-3′ and probe Sch_haem: 5’-[HEX]TGGAGACTTGTGAATGGTCGAACG[BHQ1]-3′ for *S. haematobium* and primers SchM_F: 5′-CTGCTCAGTGAAGAAGTTTGTTT-3′ and SchM_R: 5′-CCTCATTGAACCATTCACAAGTC-3′ and probe SchM_Probe: 5’-[6FAM]AGCCGCGATTATTTATCGTGCTAAGGT[BHQ1]-3′(Obeng et al., 2008). Again, this was performed on the MIC machine in a 12 μL total volume. The protocol consisted of 3 min at 95 °C followed by 40 cycles of 10 s at 95 °C and 30 s at 60 °C.

The *Fasciola* spp. screen was performed with specific probes for *F. hepatica* and *F. gigantica* using primers that amplify both species, SSCPFaF: 5′-TTGGTACTCAGTTGTCAGTGTG-3′ and SSCPFaR: 5′-AGCATCAGACACATGACCAAG-3′ with probes to specifically detect *F. hepatica* and *F. gigantica*, ProFh: 5’-[6FAM]ACCAGGCACGTTCCGTCACTGTCACTTT[BHQ1]-3′ and ProFg: 5’-[HEX]ACCAGGCACGTTCCGTTACTGTTACTTTGTC[BHQ1]-3′ respectively. The screen was performed on the samples as a real-time PCR using a MIC machine. The qPCR run consisted of 5 min at 95 °C followed by 40 cycles of 5 s at 95 °C and 30 s at 60 °C (Alasaad et al., 2011).

### 2.5 Additional snail surveys mapping further locations

In October 2023, after additional spot searching of rice paddies, snails were found at three further locations (S16.02648°, E34.81704°; S16.02989°, E34.80642° & S16.09714°, E34.83408°). Each rice paddy had separate water supplies, being within a 10 km range of each other.

## 3 Results

Upon NCBI BLAST search, our cox1 sequence (accession number: OR793173.1) matched with 98.7% identity NC018536.1, and differed by eight synonymous mutations. Deposited by Liu et al. (2012), NC018536.1 is the 13,768 bp complete mitochondrial genome of *Galba pervia* (von Martens, 1867), an accepted junior synonym of *Orientogalba ollula* (Gould, 1859). Notably, higher nucleotide similarities of 99.2% over a shorter 520 bp cox1 region were seen with 28 other GenBank entries, as exemplified by OQ974908.1, being representative of *Orientogalba viridis*.

It was noted swimming behaviour of miracidia was not altered by snail presence nor were miracidia seen to enter snail tissues during this time of observation. Snail mortality was broadly equivalent by group, ranging from 62% to 75% in exposed groups and 64% in the non-exposed group. No shedding cercaria was observed. For schistosomiasis, 15% of field-caught (*non-exposed*) and 35% of field-caught (*exposed*) snails appeared with tangible evidence of *Schistosoma* DNA with Ct values great than expected for background levels*,* especially as those snails that died shortly after exposure, exhibited the lowest C_t_ (28–32) values. Application of species-specific probes for fascioliasis provided no evidence of pre-patent infection.

## 4 Discussion

We remain mindful that taxonomy and systematics within the Lymnaeidae can be contentious, we discern that “*Lymnaea viridis*” was first described over 150 years from Guam and is now placed within the genus *Orientogalba* Kruglov and Starobogatov, 1985. As populational sampling increases, DNA evidence and phylogenetic analyses accumulate, we expect that *O. ollula* will be found conspecific with *O. viridis*, becoming another junior synonym. Hence, we are confident that our encountered snail population is best described as *O. viridis*. The temporal origin(s) of this snail locally is open to debate, as its associated rice paddies first came into production decades ago, it may well have gone unnoticed since then (see below).

This is not our first time to have encountered an alien lymnaeid in Malawi. In 2023, another alien lymnaeid species *Pseudosuccinea columella* (Say, 1815) was first encountered in Mangochi, Chikwawa and Nsanje Districts. This species is a well-known intermediate snail host for both human and animal fascioliasis (Jones et al., 2024) and the newly recognized presence of this alien intermediate snail and *O. viridis* flag a fresh concern in altered local transmission potential for human and animal fascioliasis. As noted by Schniebs et al. (2017), *O. viridis* was very clearly associated with rice paddies. Indeed, these amphibious snails can be found in very large numbers in between mature rice plants upon shady moist soil, and only very rarely upon emergent vegetation within fringing ponds. It is typically in these ponds where intermediate snail hosts for schistosomaisis, *Bulinus* spp., are dominant. Once the rice paddy starts to dry out, as plants mature, these particular intermediate hosts are absent, being unable to survive drying out, especially if unable to aestivate by burying into the mud (Brown, 1994). The latter has important implications for risk of schistosomiasis transmission locally. For example, rice paddies are tended only by a handful of people whereas fringing communal ponds are extensively used, especially by children, for bathing and swimming, see location photograph in Fig. 1. In Chikwawa District, subsistence rice farming first started here some 20 years ago. Whilst farmers were aware of this peculiar snail, seen during hand-tilling and twice-a-year cropping, they were unaware of its alien intermediate host nature. An absence of fascioliasis within these inspected populations is consistent with local practices of keeping livestock away from paddies to protect rice seedlings.

We surmise that *Orientogalba viridis* has been recently introduced in Malawi and that this species is also a new mollusc for the African continental fauna. It could represent a founder population for further spread into the African hinterland. This might easily be accomplished by further transfer of rice plants as has likely happened across these separated rice paddies in Chikwawa District. Its dispersion could be aided by cyclone events that promote wider flooding, distributing snails, alongside other movements on birds (Figuerola and Green, 2002; Boag 1986).

Although no trematode larvae were detected in the observed specimens by microscopy, molecular xenomonitoring has provided evidence to suggest that there may be some proliferation of *Schistosoma* sp. within experimentally infected snails. If these experimental challenges were performed on a greater number of snails, and with higher survival rates, alongside a much longer period of pre-patent scrutiny (i.e. > 45 days), we might well see a snail develop their experimental infection onto successful cercariogenesis. The observed *Schistosoma* DNA C_t_ values that were lower in exposed snails versus field caught snails, which were notably quite higher partially corroborates. We postulate that only a few *O. viridis*, if able to survive the pre-patent developmental period, perhaps may go onto shed cercariae, albeit in low numbers.

Our conjecture on experimental infection is congruent with the singular original observation of a *S. haematobium* cercaria within our first screen of field-caught snails. To date, although no lymnaeid snail has been found naturally or demonstrated experimentally to transmit human schistosomes in Africa, lymnaeid snails in Asia are responsible for transmission of other schistosome species within the *Schistosoma indicum* group. Indeed, one member of this group, *Schistosoma incognitum* Chandler, 1926 is exclusively transmitted by a lymnaeid intermediate host snail (Lockyer et al., 2003). Until proven otherwise through additional experimental challenges, the transmission potential of *O. viridis* should not be overlooked.

Whilst it may have a possible role in carrying an infection through to cercariogenesis, other more common intermediate host snails for African schistosomiasis here in Malawi typically shed several hundred cercariae per day. This would completely supplant any new risk that *O. viridis* might pose. Indeed, human urogential schistosomiasis is highly endemic locally (Poole et al., 2014) but we resolve that *O. viridis* is of special and peculiar interest here for alien transmission of trematodiases in Malawi. More broadly, we therefore strongly advocate for increased awareness and raised vigilance for this invasive alien snail species. The latter should also make good use of molecular xenomontioring used in conjunction with experimental infection(s) as performed at larger scales.

## 5 Conclusion

In consideration of all evidence presented here, we conclude that *O. viridis* has been formally observed for the first time in Malawi and in Africa more broadly. This raises a clear research interest, with minor public health concern, for alien transmission of various human and animal trematodiases.

## Ethical approval

The authors assert that all procedures contributing to this work comply with the ethical standards of the relevant national and institutional guides on the care and use of vertebrates. Experimentation was perform on invertebrate animals alone. The study was approved in the UK by the Research Ethics Committee of the Liverpool School of Tropical Medicine (LSTM), study protocol (22–028), and in Malawi by the College of Medicine Research and Ethics Committee (COMREC), study protocol P.08/21/3381. All human participants who provided infected urines were treated on site with praziquantel (40 mg/kg).

## Declaration of competing interest

All authors have participated in (a) conception and design, or analysis and interpretation of the data; (b) drafting the article or revising it critically for important intellectual content; and (c) approval of the final version.

This manuscript has not been submitted to, nor is under review at, another journal or other publishing venue.
